# The relationship between physical exercise and problematic internet use in college students: the chain-mediated role of self-control and loneliness

**DOI:** 10.1186/s12889-024-19226-x

**Published:** 2024-06-27

**Authors:** Junshuai Xu, Liuquan Tang

**Affiliations:** 1 Graduate school, Jose Rizal University, Mandaluyong, 1550 Philippines; 2 College of Liberal Studies, Chongqing City Vocational College, No. 1099, Xinglong Avenue, Yongchuan District, Chongqing, 402160 China

**Keywords:** Physical exercise, College students, Self-control, Loneliness, Problematic internet use, Chain mediation

## Abstract

**Objective:**

From the perspective of exercise psychology, to investigate the antefacts of problematic internet use (PIU) in college students, and to reveal the chain mediating effect of self-control and loneliness between physical exercise and PIU.

**Methods:**

1081 college students in Chongqing, China were investigated by Physical Activity Rating Scale (PARS-3), Self-control Scale (SCS), Loneliness Scale (UCLA), and Internet Addiction Scale (CIAS-R), and the data were statistically analyzed by SPSS25.0 and AMOS21.0 software.

**Results:**

(1) There was a significant negative correlation between physical exercise and PIU, and the former has a direct negative predictive effect on the latter. (2) Physical exercise could indirectly influence the PIU of college students through the partial mediating effect of self-control and loneliness, respectively. (3) Physical exercise could also indirectly influence PIU through the chain mediation of “self-control → loneliness”.

**Conclusion:**

Maintaining regular physical exercise can promote the improvement of self-control and the weakening of the loneliness experience of college students, and then help to prevent or alleviate PIU behavior, which is of great significance for psychological and behavioral health.

## Introduction

With the rapid development of network technology, various forms of Internet use such as smartphones, iPads, and computers have covered people’s life, study, and work. Although this provides convenience for people, it is easy to cause adverse consequences such as problematic internet use (PIU) if too much time and energy is invested for a long time. The PIU refers to excessive internet use that hurts an individual’s mental health, social adaptation, study, and work [1], and it is highly correlated with negative health status, and poor academic performance [2]. Meanwhile, PIU can lead to social adjustment problems such as poor interpersonal relationships, poor social skills, and interpersonal alienation [3], and even disrupt their daily work and rest schedule. It can also lead to mental health problems such as anxiety, depression, compulsion, hostility, paranoia, alcoholism, and suicide [4]. Among them, college students were prone to the behaviors of PIU due to factors such as sufficient time, lack of control, and easy access to the Internet [5, 6]. Therefore, based on the group of college students, it is of great value to explore the antefacts of PIU and the relationship between different variables, and reveal the risk factors and protective factors of PIU for preventing or improving the PIU of college students and thus promoting their healthy lifestyle and physical and mental health level.

### Relationship between physical exercise and PIU

Inappropriate internet use had some serious negative effects on adolescents’ brain function, academic performance, physical health, and psychological and behavioral adaptation [7], while physical exercise intervention has been proven to have a good correction effect on the internet use behavior of mild, moderate, and severe internet addiction college students [8]. Studies have shown that physical exercise has a significant negative correlation with PIU [9], and it could also negatively predict the symptoms of internet addiction among college students [10–12]. Among them, participation in high levels of physical activity was more likely than moderate and minimal physical activity to reduce the symptoms and dimensions of internet addiction (compulsion, withdrawal symptoms, tolerance, interpersonal health problems, and time management problems) in college students [13]. It can be seen that there was a close relationship between physical exercise and PIU among college students. On the one hand, physical exercise could shorten the time of internet use and effectively prevent internet overuse [14, 15]. On the other hand, physical exercise had a negative predictive effect on PIU [16], which has been confirmed to alleviate the level of internet addiction among college students by reducing their scores on compulsive internet use, internet addiction withdrawal, internet addiction tolerance, interpersonal and health problems, and time management problems [17].

### The mediating role of self-control between physical exercise and PIU

Self-control refers to the ability of individuals to restrain or restrain their desires and needs and change their habitual behavior, thinking, and attention mode [13, 18], whose importance in promoting normative and socially desired behaviors was increasingly appreciated [19, 20]. The theory of limited self-control holds that self-control needs to consume individual resources, and the lack of self-control could easily lead to addictive behaviors [21]. The relevant research on exercise psychology showed that physical exercise could positively promote the improvement of self-control [22], and both acute and chronic physical exercise could enhance self-control [23]. It was worth noting that self-control was significantly negatively correlated with PIU (such as online game addiction), and individuals with high self-control could significantly reduce the degree of online game addiction [24, 25]. Meanwhile, low self-control ability was positively correlated with problematic mobile phone use [26], suggesting that lack of self-control may be one of the causes of PIU. Further studies have found that physical exercise could enhance self-control and indirectly reduce dependence on smartphones [27, 28], such as eight weeks of football exercise could improve the self-control ability of internet addicts, thus reducing the symptoms of internet addiction [29].

### The mediating role of loneliness between physical exercise and PIU

Loneliness is a negative emotional experience, a subjective psychological experience caused by the lack of interpersonal relationships in the process of interpersonal communication, often accompanied by depression and other negative emotional experiences and spiritual emptiness [30]. From the perspective of cognitive processing theory, loneliness is a negative emotion generated by an individual’s perception of the gap between the actual interpersonal relationship and the expected interpersonal relationship, and it is a product of subjective cognitive processing [31]. Interestingly, physical exercise seems to have a positive impact on loneliness in people of different ages, such as Chen et al. [32] pointed out that physical exercise has a negative predictive effect on loneliness in the elderly group, and the level of loneliness decreases with the increase of physical exercise participation level. Among college students, physical exercise has a significant negative correlation with loneliness, and regular physical exercise could reduce loneliness experience [33]. In the group of children and adolescents, Wang et al. [34] pointed out that a three-month exercise intervention could effectively reduce the level of loneliness and promote the development of mental health. However, college students were at an important stage of developing peer relationships, pursuing autonomy and individualization, and were susceptible to loneliness [35]. According to the cognitive-behavioral model, individuals with higher feelings of loneliness were usually more inclined to overuse the internet as psychological compensation [36], and lonely people have lower self-evaluation of social skills and prefer online social communication rather than face-to-face communication, which was likely to lead to their compulsive use of the internet and PIU [37]. Moreover, a 12-week intervention involving Baduanjin and basketball sports was shown to significantly improve the loneliness experience of college students, accompanied by a decrease in problematic smartphone use [38].

### The chain mediating role of self-control and loneliness between physical exercise and PIU

In summary, physical exercise, self-control, and loneliness all seem to be antecedent variables of PIU, and physical exercise not only indirectly affects PIU through self-control [28, 29], but could also indirectly affect PIU through loneliness [38, 39]. On this basis, studies have found that there was also a significant correlation between self-control and loneliness, and self-control has a significant negative predictive effect on loneliness, which has been confirmed to varying degrees in primary school students [40] and college students [41]. In interpersonal communication, individuals with high self-control ability usually suffer less social rejection and feel less loneliness, conversely, low self-control may lead to interpersonal rejection and thus exacerbate loneliness [42], which may be related to the fact that individuals with low self-control ability often have insufficient social communication skills, and it was easy to cause loneliness in the psychological state [43]. Further study has found that loneliness plays a mediating role between self-control and PIU among college students, as shown by the fact that individuals with high self-control levels can suppress cognition and emotions caused by rejection, treat interpersonal communication rationally, reduce loneliness, and thus reduce dependence on the internet [44]. In addition, it has been mentioned above that physical exercise is one of the predictors of self-control and loneliness, can it influence the problematic Internet use of college students through the chain mediating effect of self-control and loneliness?

### Current study

Based on previous studies, the current study mainly aims to investigate the influence of physical exercise on PIU of college students, and reveal the mediating role of self-control and loneliness in the path relationship between them, to provide a theoretical basis and practical reference for preventing or reducing PIU of college students and promoting mental health. Therefore, this study proposes the following research hypothesis: H1) Physical exercise could directly and negatively predict the PIU of college students. H2) Self-control plays a mediating role between physical exercise and PIU of college students. H3) loneliness plays a mediating role between physical exercise and PIU of college students. H4) self-control and loneliness play a chain mediating role between physical exercise and PIU of college students.

## Methods

### Participants

The cross-sectional study was conducted among college students in Chongqing, China. Firstly, the numerical random method was used to randomly select four universities from all the public undergraduate colleges in Chongqing for investigation (Chongqing City Vocational College, Chongqing University of Arts and Sciences, Southwest University, and Chongqing College of International Business and Economics). Secondly, according to the proportion of full-time undergraduate students in each university, random samples were selected from our universities at a ratio of about 1:100 respectively. During the test, the questionnaire was issued, filled in, and collected on-site, and the time for participants to fill out questionnaires should not be less than 15 min (Before filling in the questionnaire, the researcher will explain the questionnaire content and precautions to the participants, and the questionnaires were filled in a quiet classroom). In this study, a total of 1287 questionnaires were collected, and 126 invalid samples (such as unknown key information, incomplete filling, random filling, and missing data) were eliminated through descriptive statistics, numerical conversion, and missing value processing. Finally, 1081 valid questionnaires were obtained, with an effective rate of 83.99%. Among them, 417 (38.58%) were registered in urban areas and 664 (61.42%) in rural areas. There were 523 male students (48.38%) with an average age of 19.97 ± 1.68 years. There were 558 female students (51.62%) with an average age of 20.11 ± 1.77 years. The study followed the Declaration of Helsinki and obtained written informed consent from all participants.

### Measurement tools

#### Demographic investigate

The demographic data of the participants were investigated, including gender, age, household registration, school, grade, and major.

#### Physical activity scale (PARS-3)

The PARS-3 of Liang [45] was used to assess participants’ physical exercise. There are three items in the scale, including exercise intensity (how much intensity do you think each time you participate in physical exercise), exercise frequency (how many times you do physical exercise per week), and exercise time (how many minutes do you participate in physical exercise each time). The Likert 5-point scoring was adopted, exercise intensity and exercise frequency were calculated as 1 to 5 points, exercise time was calculated as 0 to 4 points, and the formula “exercise intensity × exercise time × exercise frequency” was followed to quantify the total amount of physical exercise of the participants. The score range was 0 to 100 points, and the higher the score, the greater the exercise amount. In this study, the retest reliability of the scale was 0.75, the absolute values of the correlation coefficients among the three items were all less than 0.5, the factor load of each item was greater than 0.50, and the Cronbach α coefficient of the scale was 0.83. It showed that the scale has good reliability and validity in this study.

#### Revision of self-control scale (SCS)

The SCS compiled by Tangney et al. [46] and revised by Tan et al. [47] was used to assess participants’ self-control. The scale consists of 19 items (such as “I can resist temptation well”), including impulse control (6 items), healthy habits (3 items), resist temptation (4 items), focus on work (3 items), and limiting entertainment (3 items). The Likert 5-point scoring was adopted, from “completely inconsistent” to “very consistent”, the score was 1 to 5 points, and 15 items were scored in reverse. The total score of self-control was composed of 19 items, and the score range was 19 to 95 points, and the higher the score, the higher the self-control ability. In this study, the retest reliability of this scale was 0.83, and all factor loads were greater than 0.50, AVE was greater than 0.70, and the combined reliability of CR was greater than 0.70, indicating that this scale has good convergent validity. Meanwhile, the Cronbach α coefficient of the total volume table was 0.86, and the Cronbach α coefficient of the five dimensions was 0.81, 0.85, 0.90, 0.88, and 0.91, respectively. The results of the confirmatory factor analysis were as follows: x^2^/df = 1.97, RMSEA = 0.04, AGFI = 0.98, TLI = 0.96, CFI = 0.96, IFI = 0.97, and GFI = 0.99. It showed that the scale has good reliability and validity in this study.

#### Loneliness scale (UCLA)

Russell et al. [48, 49] compiled and revised UCLA to assess participants’ levels of loneliness. The scale has 20 items (“Do you often feel like no one knows you very well? “), which was a single dimension. The Likert 4-point scoring was adopted, from “never” to “always”, the score was 1 to 4 points respectively, among which 9 items were scored in reverse. The total score of loneliness was composed of the sum of 20 items, and the score range was 20 to 80 points, the higher the score, the stronger the loneliness. In this study, the retest reliability of the scale was 0.83, the factor load was greater than 0.50, the AVE was greater than 0.60, and the combined reliability CR was greater than 0.70, indicating that the scale has good convergent validity. Meanwhile, the Cronbach α coefficient of this scale was 0.82. It showed that the scale has good reliability and validity in this study.

#### Revised Chinese internet addiction scale (CIAS-R)

The CIAS-R of Bai et al. [50] was used to assess participants’ degree of internet use. The scale has 19 items (such as “I find myself spending more and more time online”), including compulsive internet use and withdrawal reaction (6 items), internet addiction tolerance (4 items), interpersonal and health problems (5 items), and time management problems (4 items). The Likert 4-point scoring was adopted, with 1–4 points from “strongly disagree” to “strongly agree”. The total score of internet addiction was formed by adding the scores of 19 items, and the score range was 19–76 points, and the higher the score, the higher the degree of internet addiction. In this study, the retest reliability of this scale was 0.81, and all factor loads were greater than 0.50, AVE greater than 0.70, and combined reliability CR greater than 0.70, indicating that this scale has good convergent validity. Meanwhile, the Cronbach α coefficient of the total volume table was 0.85, and the Cronbach α coefficient of the four dimensions was 0.90, 0.86, 0.84, and 0.88, respectively. The results of the confirmatory factor analysis were as follows: x^2^/df = 2.02, RMSEA = 0.04, AGFI = 0.96, TLI = 0.95, CFI = 0.98, IFI = 0.98, and GFI = 0.97. It showed that the scale has good reliability and validity in this study.

### Methods

The SPSS 25.0 was used to process and analyze the data in this study. The descriptive analysis, exploratory factor analysis, and confirmatory factor analysis were used to test the reliability and validity of the scale. The Pearson correlation analysis was used to investigate the correlation coefficients among variables, and the mediation effect was discussed according to the mediation effect test process proposed by Wen et al. [51], and the structural equation model was established by AMOS 21.0. The significance level of all indexes was set as *p* < 0.05.

## Results

### Common method deviation test

As this study adopts the method of investigation and the data are obtained by subjectively scoring and filling in the questionnaire by the participants, the corresponding control methods such as anonymous questionnaire measurement, positive and negative scoring, and standardized testing were adopted during the test, and the Harman single factor test was used to investigate the common method bias [52]. The results showed that there were 11 factors with feature roots greater than 1, and the variation explained by the first factor was 32.26%, which was less than the critical standard of 40%, indicating that there was no common method bias in this study.

### Correlation analysis of physical exercise, self-control, loneliness, and PIU

Pearson correlation analysis showed (Table 1) that physical exercise was significantly positively correlated with self-control (*r* = 0.39, *p* < 0.001), and negatively correlated with loneliness (*r*=-0.34, *p* < 0.001) and PIU (*r*=-0.29, *p* < 0.001). The self-control was significantly negatively associated with loneliness (*r*=-0.31, *p* < 0.001) and PIU (*r*=-0.37, *p* < 0.001). There was a significant positive correlation between loneliness and PIU (*r* = 0.41, *p* < 0.001). The correlation between the main variables reached the significance level, which provided a good basis for the subsequent mediation effect test.

**Table 1:** Correlation analysis among major variables

Variable	M ± SD	Physical exercise	Self-control	Loneliness	PIU
Physical exercise	25.47 ± 8.93	1.00			
Self-control	38.66 ± 10.35	0.39^***^	1.00		
Loneliness	41.29 ± 9.67	-0.34^***^	-0.31^***^	1.00	
PIU	44.18 ± 11.33	-0.29^***^	-0.37^***^	0.41^***^	1.00

Note: * means *p* < 0.05, ** means *p* < 0.01, *** means *p* < 0.001

### Analysis of the mediating effects of self-control and loneliness on physical exercise and PIU

According to the mediation effect test process proposed by Wen et al. [51], this study examined the pathway relationship between college students’ physical exercise, self-control, loneliness, and PIU. First, the total effect of physical exercise on PIU was examined, and then the model fit and the significance of each path coefficient were examined after adding mediating variables (self-control, and loneliness). Under the premise that the mediating effect was significant, if the direct effect of physical exercise on PIU becomes insignificant after adding the mediating variables, it indicates that there is a complete mediating effect; otherwise, there is a partial mediating effect. Moreover, gender and age are controlled in the structural equation model as covariates.

**Fig. 1:**
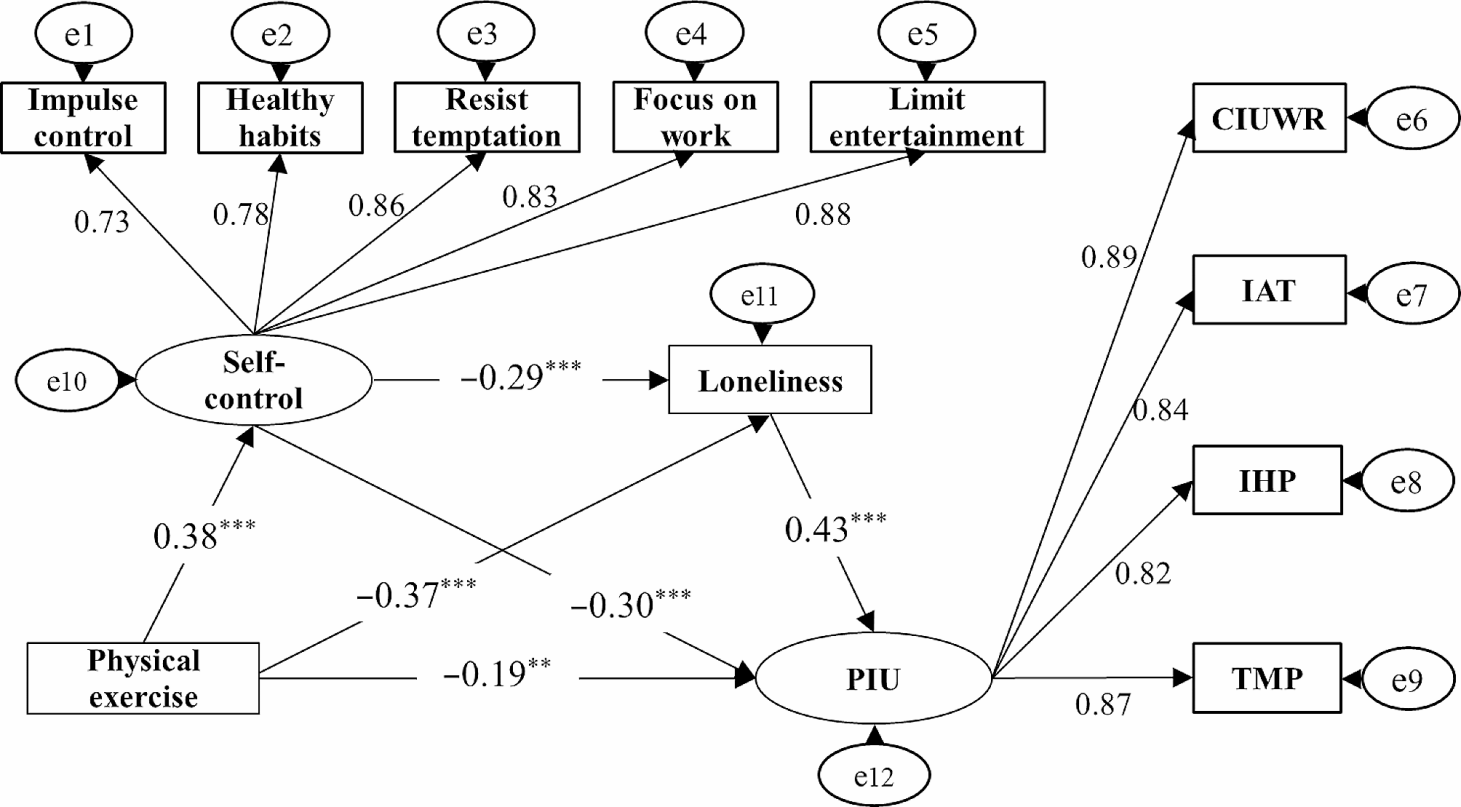
A model of the chain-mediated effects of self-control and loneliness Note: PIU: problematic internet use; CIUWR: compulsive internet use and withdrawal reaction; IAT: internet addiction tolerance; IHP: interpersonal and health problems; TMP: time management problems

The structural equation model was used to test the path relationship among variables (Fig. 1), and the fitting indexes of the model were as follows: x^2^/df = 2.09, RMSEA = 0.04, AGFI = 0.96, TLI = 0.99, CFI = 0.97, IFI = 0.97, and GFI = 0.99, indicating that the model has a good fit and was suitable for mediating effect test. The results showed that: (1) Physical exercise could directly and significantly negatively predict PIU (β1=-0.30, SE = 0.03, *p* < 0.001). After adding the mediating variables of self-control and loneliness, the path coefficient used by physical exercise on PIU decreased from β1 to β2, but the path coefficient still reached a significant level (β2=-0.19, SE = 0.01, *p* < 0.01). (2) Physical exercise could significantly positively predict self-control (β = 0.38, SE = 0.04, *p* < 0.001), and self-control could significantly negatively predict PIU (β=-0.30, SE = 0.02, *p* < 0.001). The results showed that the mediating effect of the path of “physical exercise → self-control → problematic network use” was significant, and its effect size was 0.38× (-0.30) = -0.11. (3) Physical exercise could significantly negatively predict loneliness (β=-0.37, SE = 0.02, *p* < 0.001), and loneliness could significantly positively predict PIU (β = 0.43, SE = 0.03, *p* < 0.001). The results showed that the mediating effect of “physical exercise → loneliness → PIU” was significant, and its effect size was − 0.37 × 0.43= -0.16.

Moreover, according to the research suggestion of Taylor et al. [53] on the probability and statistical power of type I errors when there was a “mediating effect with three paths”, the joint significance method was used to test the chain mediating effect of “self-control → loneliness” in the model. The results showed that self-control could negatively predict loneliness (β=-0.29, *p* < 0.001), indicating that the mediating effect of the chain path of “physical exercise → self-control → loneliness → PIU” was significant, and the effect size was 0.38× (-0.29) ×0.43=-0.05.

Judging from the influence path and effect of physical exercise on PIU (Table 2), the total effect of physical exercise on PIU was − 0.51, the direct effect was − 0.19 (accounting for the total effect ratio of 37.25%), and the total intermediary effect was − 0.32 (accounting for the total effect ratio of 62.75%). Among them, the mediating effect of self-control, loneliness, and self-control → loneliness accounted for 21.57%, 31.37%, and 9.80%, respectively, and 95% confidence intervals for each path do not contain 0. In conclusion, all the hypotheses in this study have been effectively confirmed.

**Table 2:** Influence path and effect size table of physical exercise on PIU

effect	path	Standardized effect size	Boot 95% CI	Effect ratio
Direct effect	Physical Exercise →PIU	-0.19	(-0.22, -0.16)	37.25%
Mediating effect	Physical exercise →Self-control →PIU	-0.11	(-0.13, -0.09)	21.57%
	Physical exercise →Loneliness →PIU	-0.16	(-0.19, -0.14)	31.37%
	Physical exercise →Self-control →Loneliness →PIU	-0.05	(-0.06, -0.03)	9.80%
Total mediating effect		-0.32		62.75%
Total effect		-0.51		100%

## Discussion

### Discussion of direct effects

PIU has developed into a global public health problem that cannot be ignored, and it has significant harm to individual physical and mental health [2, 4], this study confirmed that there was a linear relationship between physical exercise and PIU of college students, that is, physical exercise could directly and negatively predict PIU. This is consistent with previous studies, such as the significant negative correlation between physical exercise and problematic smartphone use [54], and it also has a direct negative predictive effect on compulsive internet behavior, internet addiction withdrawal reaction, internet addiction tolerance, interpersonal relationship and health problems, and time management problems of college students [33]. Meanwhile, physical exercise intervention could effectively reduce PIU among college students [55]. In a 16-week intervention study, researchers found that Tai Chi could alleviate the level of internet addiction of Chinese college students [17], and physical exercise intervention had a good effect on the PIU behavior of mild, moderate, and severe internet addiction college students [8]. We speculate that the direct negative predictive effect of physical exercise on PIU was related to two factors. On the one hand, physical exercise participation reduces the duration and frequency of internet use among college students. For example, it has been found that the more time spent on physical exercise, the less problematic smartphone use occurs [15], whereas moderate-to-high intensity physical activity (MVPA) was considered to be effective in preventing smartphone overuse [14]. On the other hand, physical exercise can promote the release of neurotransmitters and enhance pleasant experiences, such as it can significantly increase the plasma dopamine and β-endorphin concentrations of college students, which was conducive to the enjoyment of exercise for individuals and alleviate internet addiction [17, 56]. Therefore, regular physical exercise could replace internet use behavior to some extent and is an effective strategy to prevent or improve the PIU behavior of college students.

### Discussion on mediation effect

According to the Addictive Behavior Model (I-PACE), the reduction of individual executive control and inhibitory control leads to the reduction of motivation seeking and desire suppression, which leads to excessive addictive behavior [57], and PIU behavior was an impulsive control disorder in the internet context, and its essence was the behavior problem of individuals lacking the ability to control internet use [58]. Studies have shown that self-control could directly and negatively predict PIU of college students [44, 59], when encountering negative life events, college students with low self-control levels may be more likely to immerse themselves in negative emotions and satisfy the urge to immediately use smartphones to seek comfort, thus leading to smartphone addiction [60]. Interestingly, our results suggested that physical exercise may negatively affect PIU through the mediating role of self-control. An intensity model of self-control suggested that self-control could be effectively improved or enhanced through regular physical activity or exercise [61] and significantly improve inhibition and control deficits in individuals with smartphone addiction [62]. Meanwhile, there was a positive correlation between different types and intensity of physical exercise, and self-control [62, 63] had a positive impact on self-control [64]. For example, the study has found that the self-control ability of college students with high physical activity is usually higher than that of students with moderate physical activity, while those with moderate physical activity are usually higher than those with low physical activity [13]. This may be related to the fact that physical exercise promotes better attention distribution and greater P3 event-related potential amplitudes [65] and improves inhibitory control associated with the prefrontal cortex to mitigate addictive behaviors [66], and it has been validated effectively in drug addicts [67, 68]. Du et al. [13] also found similar findings in internet addiction groups, that is, physical exercise could indirectly affect internet addiction through the mediating role of self-control. Combined with the results of this study, we speculate that physical exercise can improve the brain areas related to inhibition control and enhance the ability to resist the impulse and behavior of excessive internet use, which was conducive to preventing or alleviating the behaviors of PIU in college students.

Loneliness reflects a deficiency in interpersonal relationships, caused by dissatisfaction with real interpersonal relationships [69]. From the perspective of social needs theory, to make up for the lack of actual interpersonal relationships and avoid dealing with the negative emotions caused by loneliness, individuals with high loneliness were more likely to overuse the internet [70]. Our results showed that loneliness was positively correlated with PIU and that it plays a mediating role between physical exercise and PIU. On the one hand, loneliness was associated with increased internet use, and lonely people may use excessive internet use as a coping strategy for seeking emotional support and social interaction [71]. Caplan [37] explained the problem of PIU from the perspective of social skills, that is, it was assumed that deeply lonely individuals will be attracted to online social interaction because they realize that they lack social skills. Therefore, they tend to use the internet for social interaction instead of face-to-face contact, resulting in PIU. On the other hand, a review of studies has shown that different types of exercise (such as single sport, team sport, double sport, and team + double sport) could alleviate internet addiction by improving the mental health of students with internet addiction (such as loneliness and anxiety) [72]. Meanwhile, Cheng et al. [39] also found that participation in physical exercise was related to the improvement and adjustment of college student’s mental health, and exercise could effectively promote the release of psychological pressure, increase social opportunities, and reduce negative emotions such as loneliness in the process of exercise, thus playing a role in relieving mobile phone addiction. It can be seen that physical exercise has the effects of regulating negative emotions and promoting social communication, and such effects can potentially prevent or improve PIU by reducing the loneliness of college students.

In addition, we established a structural equation model with “self-control → loneliness” as the chain mediation, and found that the chain mediation effect of “self-control → loneliness” between physical exercise and PIU also reached a significant level. According to self-control theory, low control means failure of control, failure of self-control hurts others, and the negative impact of people in a group will lead to an increased risk of being rejected by others, which will lead to increased loneliness [73]. Meanwhile, students with low self-control ability are usually unable to properly regulate their behaviors and emotions, so they perform worse in interaction and relationships with their peers [74], and thus tend to feel lonely. When individuals with low self-control experience rejection similar to those in the past, the original painful experience is re-experienced and the individual develops dissatisfaction with relationships, which makes them feel lonely, and then they rely on the internet to compensate for real unmet relationship needs [44]. Therefore, individuals who lack self-control were difficult to monitor and regulate their emotions and subsequent bad behaviors in a timely and effective manner, such as loneliness and PIU. In contrast, individuals with high self-control tend to experience less social rejection in interpersonal interactions, have lower levels of loneliness [42], and have fewer PIU behaviors. The research on exercise psychology showed that regular physical exercise could not only improve the self-control ability of college students [13, 61] but can also effectively improve their loneliness experience, which was accompanied by a decrease in problematic smartphone use [38]. On this basis, we confirmed that physical exercise could influence the PIU of college students through the mediating effects of self-control and loneliness, respectively, and further revealed that physical exercise could improve self-control ability, promote emotional regulation, and enhance social interaction, thus reducing the experience of loneliness, and ultimately prevent or improve the effect of PIU.

### Limitations

Based on cross-sectional investigation and research design, this study built a structural equation model of physical exercise and PIU of college students, although it could reveal the path relationship between various variables to a certain extent, it could not draw an exact causal relationship. In the future, cross-lag or experimental research design could be used to test and improve the research results. Meanwhile, the degree of internet use by college students was affected by many factors, and the selection of mediating variables in this study only included self-control and loneliness, subsequent studies can explore more mediating or moderating variables to better explore the anthems that affect internet use. Finally, this study mainly examined the relationship between physical exercise and self-control, loneliness, and PIU as a whole, and did not classify and compare the types and intensity of physical exercise of participants. Follow-up studies can classify and compare the comprehensive variable of physical exercise to explore whether exercise items will affect the research results.

## Conclusions

It was well known that physical exercise, as one of the antecedent variables, has a direct negative predictive effect on the PIU of college students. However, this study using structural equation models also confirmed the mediating role of self-control and loneliness between physical exercise and problematic Internet use, respectively. Not only that, but physical exercise can also have a significant impact on the PIU of college students through the chain mediating effect of “self-control → loneliness”, which was undoubtedly the enrichment and perfection of the field of exercise psychology. The results suggested that regular physical exercise has positive effects on improving self-control ability and reducing loneliness experience, and this effect was conducive to preventing or alleviating PIU of college students and plays an important role in promoting mental health and maintaining benign internet use behavior.

## Data Availability

No datasets were generated or analysed during the current study.
